# Heterozygous missense variants of *LMX1A* lead to nonsyndromic hearing impairment and vestibular dysfunction

**DOI:** 10.1007/s00439-018-1880-5

**Published:** 2018-05-12

**Authors:** Mieke Wesdorp, Pia A. M. de Koning Gans, Margit Schraders, Jaap Oostrik, Martijn A. Huynen, Hanka Venselaar, Andy J. Beynon, Judith van Gaalen, Vitória Piai, Nicol Voermans, Michelle M. van Rossum, Bas P. Hartel, Stefan H. Lelieveld, Laurens Wiel, Berit Verbist, Liselotte J. Rotteveel, Marieke F. van Dooren, Peter Lichtner, Henricus P. M. Kunst, Ilse Feenstra, Ronald J. C. Admiraal, DOOFNL Consortium, M. F. van Dooren, H. H. W. de Gier, E. H. Hoefsloot, M. P. van der Schroeff, S. G. Kant, L. J. C. Rotteveel, S. G. M. Frints, J. R. Hof, R. J. Stokroos, E. K. Vanhoutte, R. J. C. Admiraal, I. Feenstra, H. Kremer, H. P. M. Kunst, R. J. E. Pennings, H. G. Yntema, A. J. van Essen, R. H. Free, J. S. Klein-Wassink, Helger G. Yntema, Lies H. Hoefsloot, Ronald J. E. Pennings, Hannie Kremer

**Affiliations:** 1 Department of Otorhinolaryngology, Hearing and Genes, Radboud University Medical Center, Internal Postal Code 377, P.O. Box 9101, 6500 HB Nijmegen, The Netherlands; 2 The Radboud Institute for Molecular Life Sciences, Radboud University Medical Center, Nijmegen, The Netherlands; 3 Donders Institute for Brain, Cognition and Behaviour, Radboud University Medical Center, Nijmegen, The Netherlands; 4 Department of Clinical Genetics, Leiden University Medical Center, Leiden, The Netherlands; 5 Centre for Molecular and Biomolecular Informatics, Radboud University Medical Center, Nijmegen, The Netherlands; 6 Department of Neurology, Radboud University Medical Center, Nijmegen, The Netherlands; 7 Department of Medical Psychology, Radboud University Medical Center, Nijmegen, The Netherlands; 8 Department of Dermatology, Radboud University Medical Center, Nijmegen, The Netherlands; 9 Department of Human Genetics, Radboud University Medical Center, Nijmegen, The Netherlands; 10 Department of Radiology, Radboud University Medical Center, Nijmegen, The Netherlands; 11 Department of Radiology, Leiden University Medical Center, Leiden, The Netherlands; 12 Department of Otolaryngology, Head and Neck Surgery, LUMC, Leiden, The Netherlands; 13 Department of Clinical Genetics, Erasmus Medical Centre, Rotterdam, The Netherlands; 14 Institute of Human Genetics, Helmholtz Zentrum München, Neuherberg, Germany

## Abstract

Unraveling the causes and pathomechanisms of progressive disorders is essential for the development of therapeutic strategies. Here, we identified heterozygous pathogenic missense variants of *LMX1A* in two families of Dutch origin with progressive nonsyndromic hearing impairment (HI), using whole exome sequencing. One variant, c.721G > C (p.Val241Leu), occurred de novo and is predicted to affect the homeodomain of LMX1A, which is essential for DNA binding. The second variant, c.290G > C (p.Cys97Ser), predicted to affect a zinc-binding residue of the second LIM domain that is involved in protein–protein interactions. Bi-allelic deleterious variants of *Lmx1a* are associated with a complex phenotype in mice, including deafness and vestibular defects, due to arrest of inner ear development. Although *Lmx1a* mouse mutants demonstrate neurological, skeletal, pigmentation and reproductive system abnormalities, no syndromic features were present in the participating subjects of either family. *LMX1A* has previously been suggested as a candidate gene for intellectual disability, but our data do not support this, as affected subjects displayed normal cognition. Large variability was observed in the age of onset (a)symmetry, severity and progression rate of HI. About half of the affected individuals displayed vestibular dysfunction and experienced symptoms thereof. The late-onset progressive phenotype and the absence of cochleovestibular malformations on computed tomography scans indicate that heterozygous defects of *LMX1A* do not result in severe developmental abnormalities in humans. We propose that a single *LMX1A* wild-type copy is sufficient for normal development but insufficient for maintenance of cochleovestibular function. Alternatively, minor cochleovestibular developmental abnormalities could eventually lead to the progressive phenotype seen in the families.

**Electronic supplementary material:**

The online version of this article (10.1007/s00439-018-1880-5) contains supplementary material, which is available to authorized users.

## Introduction

Hereditary nonsyndromic hearing impairment (NSHI, MIM: 500008) is genetically very heterogeneous. Currently, more than 100 deafness genes have been identified, and still every year novel ones are discovered (Hereditary Hearing Loss Homepage). Since 2010, whole exome sequencing (WES) and targeted next generation sequencing have enabled the rapid and cost-efficient identification of deafness genes (Vona et al. 2015). However, we need to beware of seemingly causative variants that segregate in small families or occur in several unique individuals with hearing impairment (HI) by coincidence, especially in dominant NSHI. This is illustrated by the recent disqualification of *MYO1A* (MIM: 601478) as a deafness gene (Eisenberger et al. 2014; Patton et al. 2016). Ideally, unique pathogenic variants are identified in several families with a similar phenotype and not in a significant number of controls. Genetic studies are preferably supported by functional and animal studies that prove the deleterious effect of a variant and demonstrate the function of a gene in hearing. As this is often very time-consuming and expensive, (homology) protein modeling or existing data on studies in mice can be supportive in the discovery of novel deafness genes, as was recently exemplified by *KITLG* (MIM: 184745) (Zazo Seco et al. 2015) and *S1PR2* (MIM: 605111) (Santos-Cortez et al. 2016).

The identification of genetic defects underlying progressive HI and the pathomechanisms paves the way for development of therapeutic strategies. In the present study, we identified pathogenic missense variants of *LMX1A* (MIM: 600298), a gene associated with a complex phenotype in mice, including recessive deafness and vestibular defects (Bergstrom et al. 1999; Chizhikov et al. 2006; Steffes et al. 2012), as a cause of dominant NSHI and vestibular dysfunction in humans.

## Materials and methods

### Study subjects

This study was approved by the medical ethics committee of the Radboud University Medical Center and is in accordance with the principles of the World Medical Association Declaration of Helsinki. Written informed consent was obtained from all participants or their legal representatives. Medical history was obtained, using a questionnaire focusing on hearing and balance. External ear inspection and otoscopy to assess the tympanic membrane and aeration of the middle ear were performed in all subjects. Special attention was paid to possible causes of acquired HI. Pure tone audiometry was performed in a sound-treated room according to current standards. Air conduction thresholds were determined at 0.25, 0.5, 1, 2, 4, and 8 kHz in dB HL. Bone conduction thresholds were determined at 0.5, 1, 2, 4, and 8 kHz in dB HL to exclude conductive HI. HI was described according to the recommendations of the GENDEAF study group (Mazzoli et al. 2003). Individual progression of HI was calculated for each frequency with longitudinal linear regression analyses, using GraphPad Prism 6.0 (GraphPad, San Diego, CA, USA). Tympanometry was performed, and click-evoked ABR and otoacoustic emissions (OAEs) were obtained, according to current standards. Contralateral and ipsilateral acoustic reflexes were measured at 0.5, 1, 2 and 4 kHz up to loudness discomfort level. Speech perception thresholds and maximum speech recognition scores were determined using speech audiometry, which was performed in a sound-treated room with standard monosyllabic consonant–vowel–consonant Dutch word lists (Bosman and Smoorenburg 1995). Family 63136 did not participate in our clinical evaluation, but retrospective data from the affected subjects (I:2, II:2, II:3 and II:4) were analyzed.

Vestibular function was assessed in subjects II:7 and III:8 (family W15-0551) and the index case of family 63136, using electronystagmography (ENG) rotary chair stimulation and caloric irrigation testing, according to current standards. Additionally, cervical vestibular evoked myogenic potentials (cVEMP) and video head impulse tests (vHIT) were performed in subjects II:7 and III:8 of family W15-0551 to assess sacculus and vestibulo–ocular reflex (VOR) functionality, respectively.

To assess the presence of sub/infertility or neurological, cognitive or cutaneous abnormalities, subjects were screened in accordance with a predefined protocol (Supplemental Methods).

### Genetic analyses

#### WES

For WES, the exome was enriched with an Agilent SureSelect kit, version 4 or 5 (Santa Clara, CA, USA), and WES was performed on an Illumina HiSeq system by BGI Europe (Denmark) (Zazo Seco et al. 2017). As a first step, variants in genes associated with HI were selected (gene list DGD20062014) and classified according to the existing guidelines from the Association for Clinical Genetic Science and the Dutch Society of Clinical Genetic Laboratory Specialists (Wallis et al. 2013), as described previously (Zazo Seco et al. 2017). Mean ≥ 20× coverage was obtained for 96% for the enriched regions.

Variants in WES, shared by individuals II:3 and II:4 of family 63136 were filtered as follows: variation reads ≥ 5 and ≥ 20% and ≤ 90%, minor allele frequency (MAF) in in-house WES database (~ 20,000 exomes) ≤ 0.05%, MAF in EXAC ≤ 0.05%, MAF in GnomAD (Exomes non-Finnish Europeans) ≤ 0.05%, and location in exons or in introns in the 20 bps flanking the exons. Missense variants were only selected for segregation analysis if at least two of four tools predicted pathogenicity. Tools employed were CADD (predicted pathogenic if ≥ 15), SIFT (predicted pathogenic ≤ 0.05), Polyphen-2 (predicted pathogenic if ≥ 0.450) and Mutation Taster. A potential effect on splicing was predicted using the tools SpliceSiteFinder-like, MaxEntScan, NNSPLICE, GeneSplicer, and Human Splicing Finder as available in Alamut Visual (v 2.10, Interactive Biosoftware).

#### SNP genotyping (family 63136)

All members of family 63136 were genotyped with the Infinium^®^ Global Screening Array-24 v.1.0 (Illumina) according to protocols of the manufacturer. Genotypes were employed to exclude candidate variants derived from WES. Variants were regarded to be excluded if genotypes of SNPs located within 0.5 Mb both proximal and distal of the variant excluded the presence of the haplotype with the variant in at least one affected individual.

#### Sanger sequencing

PCR of fragments to be analyzed was performed using standard protocols. Primers for PCR were designed with Primer3Plus. Reference sequence identifiers, primer sequences and PCR conditions are provided in Table S3. PCR fragments were purified with ExoSAP-IT (Thermo Fisher Scientific), according to manufacturers’ protocols. For sequence analysis, the ABI PRISM BigDye Terminator Cycle Sequencing v2.0 Ready Reaction kit and the ABI PRISM 3730 DNA analyzer (Applied Biosystems, Foster City, CA, USA) were employed. Possibly deleterious effects of the identified variants were predicted with Alamut Visual version 2.7.1 (Interactive Biosoftware, Rouen, France).

#### Linkage analysis

Linkage analysis was performed by genotyping of all subjects of family W15-0551 with the 700 K SNP Global Screening Array (Illumina, San Diego, CA, USA), using the manufacturers’ protocols. Superlink online SNP 1.1 software was employed for approximate multipoint LOD score calculations and a window size of 20 SNPs was used (Silberstein et al. 2013). The inheritance pattern of HI was assumed to be autosomal dominant with a disease allele frequency of 0.001. The penetrance of the disease allele was set at 99%.

#### VNTR marker analysis

Haplotypes in the *LMX1A* region were determined by genotyping variable number of tandem repeat (VNTR) markers. Touchdown PCR was used to amplify the VNTR markers, and analyses were performed on an ABI Prism 3730 Genetic Analyzer (Applied Biosystems, Foster City, CA, USA). Marker heterozygosity, order, and genetic location were derived from the Marshfield genetic map. Alleles were assigned with GeneMapper v.4.0 software (Applied Biosystems, Foster City, CA, USA).

### Conservation in the LIM-homeodomain protein family

All ~ 600 members of the family were extracted from the SMART domain database (http://smart.embl-heidelberg.de/) and aligned. Sequence logos for the second LIM domain and the homeodomain within this family were created with WebLogo (Crooks et al. 2004).

## Results

### A de novo missense variant of LMX1A cosegregates with dominant NSHI

**Fig. 1:**
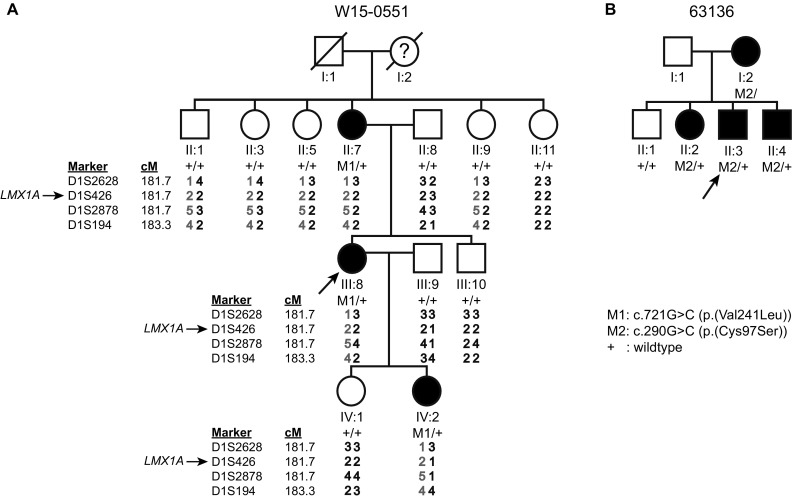
Pedigrees, VNTR genotypes and segregation of *LMX1A* variants. **a** Genotypes of VNTR markers and segregation of the identified missense variant of *LMX1A* in family W15-0551. Genetic locations of the markers were derived online from the Marshfield genetic map and maker order was confirmed in the human genome assembly GRCh37/hg19. As the variant resides on an allele (depicted in red) that is shared by non-affected siblings of subject II:7, the variant was concluded to be de novo in subject II:7. It remained unclear whether subject I:2 was affected only at high age or earlier, based on conflicting subjective information provided by her family members. **b** Pedigree and segregation analyses of a missense variant in *LMX1A* in family 63136. Index cases are indicated by arrows. + Wild type (color figure online)

A family of Dutch origin (W15-0551, Fig. 1a) was investigated to identify the underlying genetic defect of the dominant NSHI segregating in the family. We performed WES in the index case (III:8). No (likely) pathogenic variants were identified in genes known to be associated with HI. As a next step, all WES variants were analyzed. To reduce the number of candidate variants, WES was performed in subject II:7 as well. After filtering for shared and rare (≤ 0.5%), missense, nonsense, indels, and splice site variants, 186 variants remained (Table S1). One of these variants was a heterozygous missense variant of *LMX1A* at g.165179962C (GRCh37/hg19), and c.721G > C (RefSeq NM_001174069.1; p.Val241Leu) (Figure S1). This variant has not been reported in gnomAD and segregated with the HI in the family (Fig. 1a).

It was unknown whether the grandmother (subject I:2) was hearing impaired, because of conflicting subjective information provided by family members. Therefore, we investigated whether the variant of *LMX1A* was inherited or occurred de novo in subject II:7. Haplotypes of VNTR markers in the *LMX1A* region were determined. This revealed that the c.721G > C containing haplotype was also present in individuals in the second generation, who did not carry this variant (Fig. 1a). This indicates that the variant occurred de novo in subject II:7 and also that the *LMX1A* genomic region would have been excluded to carry the underlying genetic defect if linkage analysis would have been the primary step in our analysis.

**Fig. 2:**
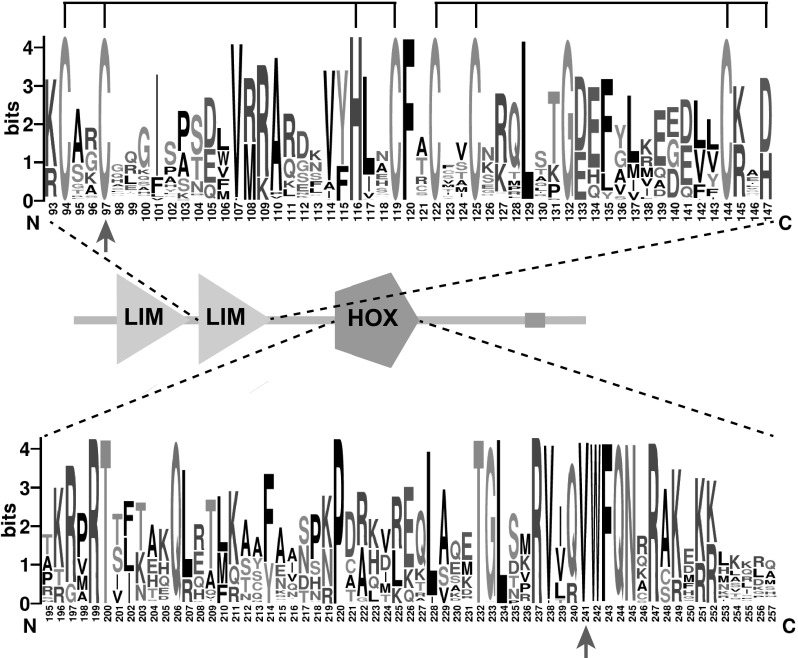
Domains and conservation of (mutated) residues in the LIM-homeodomain protein family. The presented conservation of the second LIM domain and the homeodomain shows that the mutated residues Cys97 and Val241 (indicated by arrows) are perfectly conserved. Within the second LIM domain, the residues that together bind two zinc atoms are indicated with black lines above the amino acid sequence

*LMX1A* c.721G is highly conserved (PhyloP 5.53) and p.Val241 is fully conserved in the LIM-homeodomain protein family (Fig. 2). Also, defects of *Lmx1a* have been associated with HI and vestibular defects in mouse (Bergstrom et al. 1999; Chizhikov et al. 2006; Steffes et al. 2012). All together, we considered the identified *LMX1A* variant a promising candidate to underlie the HI in family W15-0551.

To exclude other candidate DNA variants (*n* = 185) shared by individuals II:7 and III:8, linkage analysis was performed by genotyping all subjects of family W15-0551. There were 54 regions in which linkage could not be excluded by a LOD score ≤ − 2 (Figure S2, Table S2). DNA variants in these regions were filtered and classified as described above, which resulted in seven candidate variants (Table S2). None of these segregated with the HI in the family, as determined by Sanger sequencing. As ten of the 54 regions not excluded by linkage analysis harbored known deafness genes (Table S2), coverage of all exons and exon–intron boundaries of these genes, including *MYO6* (MIM: 600970), was manually checked to be at least 10×. Subsequently, Sanger sequencing was performed for regions with a lower coverage, which did not reveal any rare variants (allele frequency ≤ 0.5%). Since the HI associated with dominant defects of *MYO6* is similar to that in the affected subjects of family W15-0551 with regard to (variable) age of onset and severity (Hilgert et al. 2008; Miyagawa et al. 2015), this gene was also excluded by segregation analysis of SNPs located in and flanking this gene (Figure S3).

Rare variants in the region of chromosome 6 with the strongest evidence for linkage were evaluated for a potential deleterious effect on gene function and for the genes carrying the variants (*SLC17A5*, *PM20D1*, *MDN1*), we searched relevant databases for indications for a role in inner ear function (Table S3). All variants were intronic and none of these were predicted to affect transcript splicing (Table S3). Although *MDN1* is transcribed in the cochlea at a significant level and HI is associated with *Slc17a5* defects in mouse, it is not likely that the variants of these genes are contributing to the HI phenotype in the family or to the variability of this phenotype.

### The p.Cys97Ser amino acid substitution of LMX1A is associated with NSHI

We addressed further involvement of deleterious *LMX1A* variants in dominant NSHI. WES data of individuals with NSHI, in which (likely) pathogenic variants in all known deafness genes were excluded, were evaluated for rare variants of *LMX1A.* Only subjects with suspected dominant inheritance or without family history of HI were included (*n* = 405). This revealed a heterozygous variant at g.165218851, c.290G > C (p.Cys97Ser) in the index case of family 63136 (Figure S1), which segregated with dominantly inherited HI in the family (Fig. 1b). *LMX1A* c.290G is highly conserved (PhyloP 5.45) and variant c.290G > C has not been reported in the gnomAD database. Residue Cys97 is located within the second LIM domain, and is fully conserved in the LIM-homeodomain protein family (Fig. 2). Screening of WES data also revealed a heterozygous variant at g.165218765, c.376G > A (p.Gly126Lys) in the index case of family W05-233, but this variant was inherited from the normal hearing mother. As can be seen in Fig. 2, p.Gly126 is not conserved in the LIM-homeodomain protein family.

To address other potentially pathogenic variants in family 63136, in genes not (yet) associated with HI, WES was performed for an additional individual (II:4) of the family. Shared variants were identified and filtered as described in “Materials and methods” and SNP genotypes were employed to address potential co-segregation with the HI in the family. Based on these analyses, 15 candidate variants remained to be tested for segregation analysis by Sanger sequencing. Five of the 15 variants, all missense variants, were found to completely segregate with the HI in the family (Table S4a). However, no HI phenotype was reported for four of the five genes carrying the variants in mouse mutants (Table S4b) which does not support a causative nature of these variants for the HI in the family. For *TTYH1*, no phenotypic data were available for mouse mutants. We searched WES data of the genetically ‘unsolved’ cases with suspected dominant inheritance or without family history of HI for rare variants of this gene and did not identify any rare truncating or missense variants or variants predicted to affect splicing.

### LMX1A-associated HI is nonsyndromic, sensorineural, and progressive with a variable age of onset

The affected subjects of family W15-0551 (II:7, III:8 and IV:2) were clinically examined to characterize their audiovestibular phenotype. There was no evidence of acquired causes of HI. All affected subjects had sensorineural HI except for individual II:7 of family W15-0551, who had bilateral fenestral otosclerosis and mixed HI of the left ear. We assumed that the sensorineural HI was unlikely to be caused by otosclerosis, because there were no signs of cochlear otosclerosis on computed tomography scans and her vestibular test results fit with the defect in *LMX1A*. There were no preoperative audiograms available before stapedectomy of the right ear in 1973 for comparison of the pre- and postoperative bone conduction thresholds of the right ear.

**Fig. 3:**
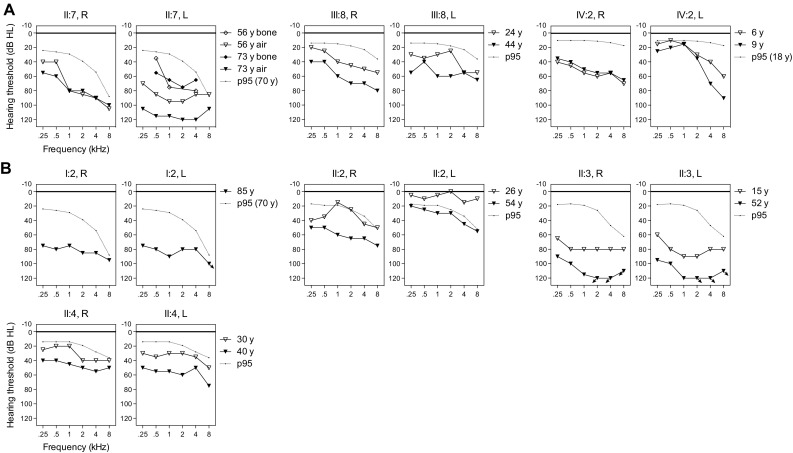
Audiometric characterization of families affected by deleterious *LMX1A* variants. Air conduction thresholds of both ears of the affected individuals are shown of first-visit and last-visit audiometry. The 95th percentile threshold values of presbyacusis (p95) were calculated for the last-visit audiogram, and matched to the individual’s sex and age, according to the ISO 7029 standard. **a** Family W15-0551. For subject II:7, also bone conduction thresholds of the left ear are depicted, because of mixed HI due to fenestral otosclerosis (conductive HI) and the *LMX1A* variant (sensorineural HI). **b** Family 63136. Subjects did not participate in our clinical evaluation; only retrospective data were used for analysis. *R* right ear, *L* left ear

The reported age of onset varied from congenital to 35 years (Table 1). Affected subjects had mild to profound HI with overall a downsloping audiogram configuration (Fig. 3). Subject IV:2 of family W15-0551 and subject II:2 of family 63136 displayed asymmetric HI, whereas the other affected individuals demonstrated symmetric HI. Longitudinal linear regression analysis of hearing thresholds revealed significant progression in all affected subjects of both families, except for subject IV:2 of family W15-0551, probably because follow-up time was too short (3 years) to measure significant deterioration of hearing in this individual. Progression of HI was not analyzed in subject II:7 of family W15-0551, as we cannot exclude deterioration of hearing due to her otosclerosis. The progression rate and severity of HI varied widely among the affected subjects (Fig. 3, Table 1).

**Table 1:** Individual results of otoscopy, pure tone audiometry, HI progression analysis, speech discrimination and vestibular complaints

Family	Subject	Age at evaluation (years)	Reported age of HI onset (years)	Otoscopic examination	PTA (dB HL)		Significant progression of HI^a^				SRT (dB)^c^	Maximum SRS (%)^c^	Vestibular complaints
					R	L	R	L	Rate^b^	YOF			
W15-0551	II:7	73	27	R + L Myringosclerosis	73	65^d^	No	No	–	17	67	58	No
	III:8	44	Puberty	R + L Hypermobile eardrum	57	53	Yes	Yes	1.2	20	51	92	Vertigo episodes at the age of 32 and 40 years lasting for days
	IV:2	9	Congenital^e^	Normal	48	23	No	No	–	3	22	100	No
63136^f^	I:2	85	Childhood	NT	80	83	–	–	–	0	82	90	No
	II:2	54	26	Normal	58	28	Yes	Yes	1.0	28	28	100	Vertigo episodes with tinnitus during puberty and at the age of 40 years lasting for days
	II:3	52	Congenital	Normal	112	113	Yes	Yes	1.1	37	ND	10	Vertigo episodes with tinnitus in the past lasting for hours
	II:4	40	35	Normal	45	57	Yes	Yes	1.6	10	36	92	Vertigo episodes with tinnitus around the age of 40 years lasting minutes to hours, and BPPV

*PTA* pure tone average, mean of 0.5, 1 and 2 kHz air conduction thresholds, *R* right, *L* left, *HI* hearing impairment, *YOF* years of follow-up, *SRT* speech reception threshold, *SRS* speech recognition score, *NT* not tested

^a^Individual progression of HI was calculated with longitudinal linear regression analyses, using all available audiograms for the better hearing ear. Audiograms were used only if they were obtained after the age of 5 years. The onset level of HI (threshold intercept, in dB HL at age 0 years) and progression of HI (slope in dB/year) were determined for each frequency (0.25, 0.5, 1, 2, 4 and 8 kHz). Progression of HI was considered to be significant, if the regression coefficient differed significantly (*p* ≤ 0.05) from 0 for at least two of the six evaluated frequencies, and if the slopes were positive. Progression of HI could not be calculated in subject I:2 of family 63136, because only one audiogram was available

^b^Progression rate is the mean (PTA_0.5–4 kHz_) increase in dB per year for the better hearing ear

^c^Results of speech discrimination for the better hearing ear are presented

^d^PTA of bone conduction thresholds is displayed, because of mixed HI due to fenestral otosclerosis conductive HI) and the *LMX1A* variant (sensorineural HI)

^e^Subject IV:2 of family W15-0551 failed the neonatal hearing screening, which indicates that she had at least 30 dB hearing loss at birth

^f^Family 63136 did not participate in our clinical evaluation; only retrospective data were analyzed

Speech reception thresholds were lower than or comparable to pure tone average thresholds at 0.5, 1 and 2 kHz for the better hearing ear, indicating the absence of retrocochlear pathology (Table 1). This is confirmed by results from acoustic reflex measurements in all affected subjects and brainstem evoked response audiometry in subject II:2 of family 63136, which showed no indications of retrocochlear pathology (data not shown). CT scans in subject II:7 of family W15-0551 and the index case (II:3) of family 63136 did not show cochleovestibular malformations, except for known fenestral otosclerosis in the former individual.

Four out of seven affected individuals reported sudden episodes of vertigo in the past that lasted minutes to days, with or without tinnitus, but without simultaneous deterioration of hearing (Table 1). Individuals could not identify triggers for the vertigo episodes. Vestibular function was assessed in subjects II:7 and III:8 (family W15-0551) and the index case of family 63136. The vestibular tests revealed bilateral symmetric hyporeflexia in subject III:8 of family W15-0551 (42-year-old), asymmetric severe hyporeflexia to areflexia to the detriment of the left vestibulum in subject II:3 of family 63136 (52-year-old), and bilateral areflexia in subject II:7 of family W15-0551 (73-year-old). Subjects II:7 and III:8 of family W15-0551 also underwent a video head impulse test (vHIT) and cervical vestibular evoked myogenic potential (cVEMP) recordings. The vHIT displayed bilateral weakness of the posterior semicircular canals in individual II:7 and normal function in individual III:8. cVEMPs showed no responses up to 100 dBnHL in both subjects, indicating dysfunction of the saccule. An overview of vestibular test results is presented in Table S6. Besides standard vestibular tests, subjects also underwent oculomotor testing, consisting of smooth pursuit, gaze (frontal/right/left), saccade, optokinetic and spontaneous nystagmus tests. Oculomotor testing revealed no abnormalities (data not shown). Since vestibular abnormalities are more severe in the older individuals, we hypothesize that there is progressive deterioration of vestibular function. There were, however, no longitudinal data available to confirm this.

As mice with bi-allelic loss-of-function variants of *Lmx1a* display neurological, skeletal, pigmentation, and reproductive system abnormalities, besides HI and vestibular dysfunction (Bergstrom et al. 1999; Chizhikov et al. 2006; Steffes et al. 2012), the affected subjects of families W15-0551 and 63136 were screened for syndromic abnormalities (Supplemental Methods). However, none of the affected individuals of families W15-0551 and 63136 displayed any cutaneous abnormalities, signs of cognitive dysfunction, or peripheral or central nervous system involvement. History taking did not indicate fertility problems.

## Discussion

This study provides evidence that LMX1A is involved in hearing and vestibular function. In two families of Dutch origin, mono-allelic deleterious variants of *Lmx1a* were found to be associated with a progressive phenotype. HI started between birth and the age of 35 years, and severity and progression rate varied widely between affected individuals. About half of the affected individuals displayed vestibular dysfunction and experienced symptoms thereof. Apart from the vestibular abnormalities, the observed HI phenotype is similar to that of DFNA7, for which the underlying defect has been localized to chromosome 1q21–q23 encompassing *LMX1A* (Fagerheim et al. 1996). Therefore, *LMX1A* is a candidate gene for DFNA7. Evaluation of rare variants segregating with the HI in family 63136 revealed no strong candidates for being causative of the HI other than the described *LMX1A* variant.

*LMX1A* is a transcription factor that belongs to the highly conserved LIM-homeodomain protein family. LIM-homeodomain proteins are characterized by two cysteine-rich zinc-binding LIM motifs that are known to be involved in protein–protein interactions (Kadrmas and Beckerle 2004), and a homeodomain that is known to bind DNA (Hobert and Westphal 2000). The mutated *LMX1A* residues identified in this study are located within the second LIM domain and the homeodomain. The p.Cys97Ser affects one of the two zinc-binding residues, which is highly likely to be deleterious for protein folding and function. LIM-homeodomain transcription factors play a pivotal role in various developmental processes, and can be divided into subgroups based on sequence similarities. The vertebrate Lmx subgroup consists of paralogs *LMX1A* and *LMX1B* that have identical homeodomain sequences (Figure S4) (Hobert and Westphal 2000).

Bi-allelic loss-of-function variants of the murine *Lmx1a* gene cause congenital deafness, vestibular defects, and neurological, skeletal, pigmentation, and reproductive system abnormalities (Bergstrom et al. 1999; Chizhikov et al. 2006; Steffes et al. 2012). However, no abnormalities in addition to inner ear dysfunction were observed in the studied families. In contrast to humans, there is no auditory phenotype in heterozygous *Lmx1a* mouse mutants, but occasionally mild pigmentation abnormalities are seen (Patrylo et al. 1990; Steffes et al. 2012). Normal hearing thresholds have been measured up to 16 weeks (Steffes et al. 2012) and 3 months (unpublished data, kindly provided by Dr. Johnson, Jackson Laboratory, Bar Harbor, Maine, concerning heterozygous *Lmx1a*^*dr*-*J*^ mice). Interestingly, the genetic defect in the well-studied mouse model *Lmx1a*^*dr*-*J*^ is p.Cys82Tyr and affects one of the zinc-binding cysteine residues of the LIM1 domain (Millonig et al. 2000), which supports the pathogenicity of the identified p.Cys97Ser variant.

Heterozygous loss-of-function variants of *LMX1B* (MIM: 602575), a paralog of *LMX1A,* have been associated with nail–patella syndrome in human (NPS, MIM: 161200) (Dreyer et al. 1998; McIntosh et al. 1998; Vollrath et al. 1998). NPS is characterized by dysplastic nails, absent or hypoplastic patellae, elbow dysplasia, and iliac horns, and may be accompanied by nephropathy and/or glaucoma (Mimiwati et al. 2006; Sweeney et al. 2003). A study by Bongers et al. (2005) indicated that late-onset sensorineural HI can also be associated with defects of *LMX1B* (Bongers et al. 2005). There are striking similarities between the identified genetic defects of *LMX1A* and those of *LMX1B*. The mutated residues Cys97 and Val241 of LMX1A are in the sequence alignments at the same positions as the amino acids Cys118 and Val265 of LMX1B, respectively (Figure S4). Amino acid variant p.Val265Leu of LMX1B is known to be disease-causing and displays the same substitution as the *LMX1A* p.Val241Leu variant (Dunston et al. 2004). The equivalent of *LMX1A* variant p.Cys97Ser has not been identified in *LMX1B*, but other substitutions at this position that would also impair the binding of a zinc atom, p.Cys118Phe (Vollrath et al. 1998) and p.Cys118Tyr (Clough et al. 1999), and the equivalent cysteine–serine substitution in LIM domain 1 (p.Cys59Ser), have been associated with NPS. Resemblance of the identified *LMX1A* variants with disease-causing variants in *LMX1B* supports the former’s pathogenicity.

We hypothesize that the identified deleterious *LMX1A* variants lead to haploinsufficiency, which is also the proposed pathogenic mechanism of NPS, caused by variants of *LMX1B* (Bongers et al. 2008; Dreyer et al. 2000; Sato et al. 2005). A number of LMX1B variants have been tested for a dominant-negative effect, including p.Val265Leu, the equivalent of LMX1A p.Val241Leu, but no inhibitory effect on wild-type protein function was found (Dreyer et al. 2000; Sato et al. 2005). Haploinsufficiency of *LMX1A* might also explain the sensorineural HI in a subject with a heterozygous interstitial 1q23.3q24.1 deletion encompassing *LMX1A* (patient 9 in Chatron et al. 2015). However, the absence of an HI phenotype in mice heterozygous for loss of function variants of *Lmx1a* is not supportive of haploinsufficiency as the disease mechanism.

Our data do not support the suggested involvement of *LMX1A* in ID (Chatron et al. 2015; Mackenroth et al. 2016), as affected subjects of families W15-0551 and 63136 displayed normal cognition. Based on our findings and those of Chatron et al. (2015) and Mackenroth et al. (2016), we recommend screening for HI in subjects with mono-allelic loss of *LMX1A*.

In mouse, *Lmx1a* expression in the inner ear starts at the otocyst stage (from E10.5 onwards) and is later restricted to non-sensory epithelia of the developing cochlea and vestibular system (Huang et al. 2008; Koo et al. 2009; Nichols et al. 2008; Steffes et al. 2012). Bi-allelic *Lmx1a* defects lead to disorganization of the inner ear and lack of differentiation and separation of sensory, non-sensory and neurogenic domains of the vestibulocochlear system. As a result, *Lmx1a* mouse mutants have a short and malformed cochlear duct, no endolymphatic duct and sac, no semicircular canals, and the sacculus and utriculus remain rudimentary (Koo et al. 2009; Nichols et al. 2008; Steffes et al. 2012). Hair cells in the basal part of the cochlea display severe disorganization, whereas hair cells in the apical turn are only mildly disorganized (Nichols et al. 2008). Although expression of *Lmx1a* in the inner ear is reduced from E16.5 onwards (Huang et al. 2008), Lmx1a has been suggested to function in the maintenance of hair cells, as there is progressive hair cell loss in adult mutant mice (Nichols et al. 2008).

There is large phenotypic variability within families W15-0551 and 63136, regarding age of onset (a)symmetry, severity and progression rate of HI. Large intrafamilial phenotypic variation has also been reported for NPS (Ghoumid et al. 2016). The variable HI phenotype suggests involvement of environmental and/or genetic factors in the expression of the phenotype. The expression level of the wild-type *LMX1A* allele might well be one of the genetic factors. The overall downsloping audiogram configuration observed in the affected subjects corresponds to the abnormal development of the sensory epithelium in the organ of Corti in homozygous *Lmx1a* mouse mutants, displaying severe abnormalities of this epithelium in the basal part of the cochlea compared to mild defects in the apical regions (Koo et al. 2009).

The onset of HI caused by *LMX1A* defects was in the second or third decade in most of the cases, which suggests that one *LMX1A* copy is sufficient for normal cochlear development. As homozygous *Lmx1a* mutant mice displayed loss of cochlear hair cells in the adult stage, the gene has been suggested to be essential for long-term maintenance of hair cells (Nichols et al. 2008). It is therefore tempting to speculate that in the families the deterioration of HI over time is due to progressive loss of hair cells. Since vestibular complaints occurred during adulthood and vestibular dysfunction seemed progressive, we speculate that LMX1A is also critical for maintenance of the adult vestibular organs. Interestingly, it has been demonstrated that both *Lmx1a* and *Lmx1b* not only function in the developing mouse brain, as expression of both genes was found to be sustained in the adult midbrain and to be essential for survival of adult dopaminergic neurons in the midbrain (Doucet-Beaupre et al. 2016). A role in the regulation of genes with a mitochondrial function was indicated. It remains to be determined whether LMX1A, possibly in concert with LMX1B, has a similar function in the adult inner ear. So far, there are no indications for *LMX1A*/*Lmx1a* expression in the adult inner ear (Huang et al. 2008; Schrauwen et al. 2016). However, the expression might well be at a very low level and/or in a specific cell type. An alternative explanation for the HI in the presented families is that a single copy of *LMX1A* causes minor cochleovestibular developmental abnormalities that eventually lead to a progressive disease-phenotype. The phenotype of bi-allelic deleterious *LMX1A* variants in humans is still elusive. As suggested by Steffes et al. (2012), bi-allelic loss-of-function variants of *LMX1A* might well be lethal in human (Steffes et al. 2012).

One of the two *LMX1A* variants identified in this study occurred de novo. Also, we identified de novo variants with a dominant effect to explain four of 67 cases for whom a genetic diagnosis was established by testing a deafness gene panel and Kasakura-Kimura et al. (2017) identified de novo variants as the cause of dominantly inherited HI in two of 74 ‘solved’ cases (Kasakura-Kimura et al. 2017; Zazo Seco et al. 2015). Interestingly, also for retinitis pigmentosa that displays very high genetic heterogeneity as does HI, the underlying genetic defect was found to be a de novo variant in 3 of 28 individuals without family history of the disease (Neveling et al. 2012). These data demonstrate that in families with an isolated case of a genetically highly heterogeneous disorder for which reproductive fitness is not reduced, the scenario of an underlying de novo variant is not uncommon.

In conclusion, mono-allelic missense variants of *LMX1A* were found to underlie nonsyndromic HI and vestibular defects. The HI phenotype has a variable age of onset, severity and progression rate. Haploinsufficiency is the most likely pathogenic mechanism. As in *LMX1B,* we expect that both truncating and missense variants can lead to the identified phenotype, with a preferential presence of missense variants in the conserved residues of the LIM domains and homeodomain of *LMX1A*.

## Web resources

The URLs for data presented herein are as follows:

Alamut Visual http://www.interactive-biosoftware.com/alamut-visual/.

ExAC Browser http://exac.broadinstitute.org/.

GnomAD browser http://gnomad.broadinstitute.org/.

Hereditary Hearing loss Homepage http://hereditaryhearingloss.org/.

Marshfield Genetic Maps http://research.marshfieldclinic.org/genetics/GeneticResearch/compMaps.asp.

OMIM http://www.omim.org/.

OMIM Phenotypic Series http://www.omim.org/phenotypicSeriesTitle/all.

Primer3Plus http://www.bioinformatics.nl/cgi-bin/primer3plus/primer3plus.cgi.

SMART http://smart.embl-heidelberg.de/.

Superlink Online SNP http://cbl-hap.cs.technion.ac.il/superlink-snp/.

UCSC Genome Browser https://genome.ucsc.edu.

WebLogo http://weblogo.berkeley.edu/.

## Electronic supplementary material

Below is the link to the electronic supplementary material.

Supplementary material 1 (DOCX 3548 kb)
