# Supplementary material for: Heterozygous missense variants of LMX1A lead to nonsyndromic hearing impairment and vestibular dysfunction
Source: Hum Genet. 2018 May 12;137(5):389–400. doi: 10.1007/s00439-018-1880-5 (PMC5973959; doi:10.1007/s00439-018-1880-5)
Supplement: Supplementary file 1 — Supplementary material 1 (DOCX 3548 kb) [file 439_2018_1880_MOESM1_ESM.docx]

**Supplemental Data**

**Heterozygous missense variants of *LMX1A* lead to nonsyndromic hearing impairment and vestibular dysfunction**

**Human Genetics**

Mieke Wesdorp^1,2,3^, Pia A.M. de Koning Gans^4^, Margit Schraders^1,3^, Jaap Oostrik^1,3^, Martijn A. Huynen^5^, Hanka Venselaar^5^, Andy J. Beynon^1^, Judith van Gaalen^3,6^, Vitória Piai^3,7^, Nicol Voermans^3,6^, Michelle M. van Rossum^8^, Bas P. Hartel^1,3^, Stefan H. Lelieveld^2,9^, Laurens Wiel^2,4,9^, Berit Verbist^10,11^ Liselotte J. Rotteveel^12^, Marieke F. van Dooren^13^, Peter Lichtner^14^, Henricus P.M. Kunst^1^, Ilse Feenstra^9^, Ronald J.C. Admiraal^1^, DOOFNL Consortium, Helger G. Yntema^3,9^, Lies H. Hoefsloot^13^, Ronald J.E. Pennings^1,3,15^, Hannie Kremer^1,3,9,15^

^1^Department of Otorhinolaryngology, Hearing & Genes, Radboud university medical center, Nijmegen, The Netherlands; ^2^The Radboud Institute for Molecular Life Sciences, Radboud university medical center, Nijmegen, The Netherlands; ^3^Donders Institute for Brain, Cognition and Behaviour, Radboud university medical center, Nijmegen, The Netherlands; ^4^Department of Clinical Genetics, Leiden University Medical Center, Leiden, The Netherlands; ^5^Centre for Molecular and Biomolecular Informatics, Radboud university medical centre, Nijmegen, The Netherlands; ^6^Department of Neurology, Radboud university medical center, Nijmegen, The Netherlands; ^7^Department of Medical Psychology, Radboud university medical center, Nijmegen, The Netherlands; ^8^Department of Dermatology, Radboud university medical center, Nijmegen, The Netherlands; ^9^Department of Human Genetics, Radboud university medical center, Nijmegen, The Netherlands; ^10^Department of Radiology, Radboud university medical center, Nijmegen, The Netherlands; ^11^Department of Radiology, Leiden University Medical Center, Leiden, The Netherlands; ^12^Department of Otolaryngology, Head and Neck Surgery, LUMC, Leiden, The Netherlands; ^13^Department of Clinical Genetics, Erasmus Medical Centre, Rotterdam, The Netherlands; ^14^Institute of Human Genetics, Helmholtz Zentrum München, Neuherberg, Germany.

^15^These authors contributed equally.

Corresponding author:

Prof. dr. H. Kremer

hannie.kremer@radboudumc.nl

**Supplemental Methods**

**Screening protocol for syndromic abnormalities in subjects with *LMX1A* variants**

Medical history was obtained from all affected subjects of families W15-0551 and 63136, regarding neurological, cognitive and dermatological abnormalities, and sub/infertility. Special attention was paid to symptoms related to ataxia. Participants of family W15-0551 also underwent physical neurological and dermatological examination.

Neurological examination included testing of:

- Mental status, language and articulation
- Cranial nerves
- Muscle strength using the Medical Research Council (MRC) scores (0-5) of 18 predefined muscle groups, including shoulder abduction, elbow flexion, wrist extension, hip flexion, knee extension, foot dorsiflexion (extension) and plantar flexion, and toe dorsiflexon (extension); muscle tone and bulk
- Sensory function including pain and light touch sensation, vibration sense and position sense (absent; reduced; normal)
- Coordination with use of nose-finger test, diadochokinesis, heel-shin slide, and tandem gait (normal / abnormal)
- Deep tendon reflexes (biceps, brachioradialis, triceps, patellar, ankle jerks and plantar reflex) bilaterally (absent / reduced / normal)
- Posture and gait, including heel and toe walking (normal / abnormal)
- SARA Score (Scale for the Assessment and Rating of Ataxia)(Schmitz-Hubsch et al. 2006)

Cognitive screening of II:7 and III:8 included:

- Montreal cognitive assessment(Nasreddine et al. 2005)
- National adult reading test(Schmand et al. 1992)
- Rey auditory verbal learning test(Schmidt 1996)

Cognitive screening of IV:2 included:

- Wechsler Intelligence scale for children, similarities subset(Wechsler 1991)
- Wechsler Intelligence scale for children, coding subtest(Wechsler 1991)
- Beery-Buktenice developmental test of visual motor integration, visual motor integration subset(Beery et al. 2010)
- Rey auditory verbal learning test

Dermatological screening included evaluation of skin type (I–VI), skin depigmentation, hypopigmentation, and hyperpigmentation, number of naevi, allergic reactions, other observed skin abnormalities, and treatment of skin abnormalities in the past. The whole body was examined and a Wood’s lamp was used to analyze hypo-, hyper-, and depigmentations. If necessary, skin abnormalities were reviewed with a dermatoscope.

**Supplemental Figures and Tables**

**Figure S1. Sequences of *LMX1A* c.721G>C and c.290G>C**

Analysis of the WES paired-reads demonstrated a heterozygous missense variant in the index cases of family W15-0551, c.721G>C (p.Val241Leu) (**A**) and of family 63136, c.290G>C (p.Cys97Ser) (**B**). NM_177398.3 was employed as reference sequence. Figures show reverse sequences and were obtained using Alamut Visual version 2.7.1 (Interactive Biosoftware, Rouen, France).

**Figure S2. Genome wide LOD scores per chromosome of family W15-0551**

Linkage analysis was performed in family W15-0551 and LOD scores were calculated using SuperLink online SNP 1.1 software. The blue lines define the customary LOD score range, with a LOD score of ≥3.3 indicating significant linkage and a LOD score <-2 indicating exclusion of linkage. The green line indicates the maximum LOD score (2.4) measured in family W15-0551. In the calculations, window size was set at 20 SNPs, disease allele frequency at 0.001 and penetrance at 99%. There were 54 regions in which linkage could not be excluded (LOD score ≥ -2), but none of them had a significant maximum LOD score. The region with the highest LOD score of 2.4 was located on chromosome 6 and delimited by rs16883199 and rs2143437 (chr6:73,739,831-96,400,830; GRCh37/hg19).

**Figure S3. Haplotype analysis of the genomic region harboring *MYO6***

*MYO6* haplotypes were determined by genotyping SNPs in and flanking *MYO6* using Sanger sequencing and WES data of family W15-0551. As subject IV:2 does not inherit the grandmaternal haplotype, the causative variant does not reside in the coding or regulatory regions of *MYO6.* Black arrow indicates the index case.

**Figure S4. Alignment of LMX1A and LMX1B**

Protein alignment of LMX1A (NP_796372.1) and LMX1B (NP_002307.1) performed with Clustal Omega version 1.2.4 (EMBL-EBI, http://www.ebi.ac.uk/Tools/msa/clustalo/). An asterisk (*) indicates positions which have a single, fully conserved residue; a colon (:) indicates conservation between groups of strongly similar properties, a period (.) indicates conservation between groups of weakly similar properties. There is a high percentage of similarity between the two proteins and the homeodomain of the two proteins is identical. The mutated residues identified in LMX1A (Cys97 and Val241) are boxed and indicated by a red arrow head, and demonstrate that the amino acids at these positions are identical in LMX1B (Cys118 and Val265, respectively). LIM, LIM domain; HOX, homeodomain.

**Table S1. Filter steps applied on variants identified in WES for family W15-0551**

| Filter steps | No. of variants |
| --- | --- |
| Shared by subjects II:7 and III:8 | 85798 |
| ≤0.5% in ExAC, dbSNP database and in-house database* | 1039 |
| Exonic missense, nonsense, indels, and splice site variants** | 186 |

*in-house database contained WES data of 13314 individuals, the vast majority of Dutch origin, affected by a large number of different diseases (including 810 subjects with HI) and also non-affected individuals. **Splice site variants up to +8/-20 nucleotides were selected.

**Table S2. Overview of chromosomal regions, and WES variants in these regions, with a LOD score ≥ -2 in family W15-0551**

| **Start SNP** | **End SNP** | **Chr** | **Start position** | **End position** | **Size (Mb)** | **Max LOD score** | **Known deafness genes** | **Shared exome variants^a^** | **Rare variants^b^**  **(≤ 0.5%)** | **Coding and splice site variants** | **VUS or (likely) pathogenic variants^c^** | **Candidate variants** |
| --- | --- | --- | --- | --- | --- | --- | --- | --- | --- | --- | --- | --- |
| rs4908619 | rs2797685 | 1 | 7,369,931 | 7,879,063 | 0,51 | 0.3683 | - | 26 | 1 | 0 | - | - |
| rs12402233 | rs10875048 | 1 | 96,144,365 | 97,595,033 | 1,45 | -1.3532 | *-* | 2 | 0 | - | - | - |
| rs1470436 | rs4434842 | 1 | 102,253,545 | 104,492,118 | 2,24 | -1.0535 | *COL11A1* | 49 | 1 | 0 | - | - |
| rs478093 | rs12129861 | 1 | 120,255,126 | 145,725,689 | 25,47 | -1.2788 | - | 195 | 0 | - | - | - |
| rs10918345 | rs12041287 | 1 | 166,065,145 | 167,052,988 | 0,99 | -1.5703 | - | 8 | 0 | - | - | - |
| rs4233164 | rs10800771 | 1 | 175,651,786 | 201,240,182 | 25,59 | 1.0027 | - | 607 | 3 | 1 | 1 | *NR5A2* c.1570G>A |
| rs10920621 | rs10751435 | 1 | 203,335,113 | 205,318,321 | 1,98 | -1.6129 | - | 152 | 0 | - | - | - |
| rs1372165 | rs10153860 | 2 | 41,420,957 | 42,722,516 | 1,31 | -1.1611 | - | 13 | 0 | - | - | - |
| rs34136947 | rs115417046 | 2 | 46,584,059 | 68,108,933 | 21,52 | 0.5203 | *PNPT1* | 397 | 8 | 2 | 2 | *NRXN1* c.342G>C  *PEX13* c.89T>C |
| rs17697617 | rs16838302 | 2 | 193,564,025 | 196,067,526 | 2,50 | -1.6675 | - | 0 | - | - | - | - |
| rs1374360 | rs2348129 | 2 | 200,125,150 | 201,644,232 | 1,52 | -1.7975 | - | 31 | 1 | 0 | - | - |
| rs17609761 | rs4588514 | 4 | 48,496,804 | 54,074,642 | 5,58 | -1.6693 | - | 40 | 0 | - | - | - |
| rs621896 | rs5011719 | 6 | 8,269,415 | 22,138,150 | 13,87 | 0.3627 | - | 226 | 2 | 0 | - | - |
| rs16883199 | rs2143437 | 6 | 73,739,831 | 96,400,830 | 22,66 | 2.3974 | *MYO6* | 388 | 3^d^ | 0 | - | - |
| rs76359180 | rs4947084 | 6 | 108,810,769 | 110,947,440 | 2,14 | -1.7656 | *CD164* | 121 | 5 | 2 | 1 | *CD164* c.106-6dup |
| rs6912287 | rs72977538 | 6 | 135,597,263 | 136,736,714 | 1,14 | -0.5684 | - | 21 | 0 | - | - | - |
| rs34261503 | rs2813487 | 6 | 138,240,225 | 152,466,582 | 14,23 | 0.8980 | *-* | 290 | 2 | 2 | 0 | - |
| rs2296254 | rs1902064 | 6 | 152,555,112 | 156,722,390 | 4,17 | -1.4288 | - | 107 | 1 | 0 | - | - |
| rs17863123 | rs112640327 | 7 | 90,111,371 | 91,863,363 | 1,75 | -1.5439 | - | 16 | 1 | 1 | 1 | *FZD1* c.1571G>C |
| rs2108225 | rs17157870 | 7 | 107,453,103 | 110,287,125 | 2,83 | -0.1203 | - | 58 | 0 | - | - | - |
| rs10949787 | rs16868174 | 7 | 155,720,971 | 159,138,663 | 3,42 | 0.4142 | - | 146 | 2 | 0 | - | - |
| rs7040084 | rs13290342 | 9 | 29,483,651 | 30,175,188 | 0,69 | -1.7456 | - | 0 | - | - | - | - |
| rs117403619 | rs10511880 | 9 | 30,652,864 | 31,540,903 | 0,89 | -1.3325 | - | 0 | - | - | - | - |
| rs10970585 | rs13301121 | 9 | 31,895,373 | 34,701,352 | 2,81 | -0.8431 | - | 134 | 1 | 0 | - | - |
| rs10119417 | rs6560420 | 9 | 38,686,734 | 71,512,668 | 32,83 | -0.7518 | - | 4 | 0 | - | - | - |
| rs77600772 | rs12340637 | 9 | 77,299,919 | 78,185,039 | 0,89 | -0.1230 | - | 34 | 2 | 0 | - | - |
| rs12551266 | rs12551927 | 9 | 89,986,101 | 90,772,863 | 0,79 | -1.8168 | - | 38 | 0 | - | - | - |
| rs602924 | rs11243915 | 9 | 133,371,501 | 135,675,305 | 2,30 | 0.4537 | - | 196 | 0 | - | - | - |
| rs117496585 | rs2511376 | 11 | 91,771,186 | 93,670,388 | 1,90 | -1.3298 | - | 70 | 3 | 1 | 0 | - |
| rs74896377 | rs74536307 | 11 | 96,771,009 | 98,955,361 | 2,18 | 0.3051 | - | 0 | - | - | - | - |
| rs4282990 | rs2014365 | 11 | 124,386,011 | 125,049,965 | 0,66 | -1.8146 | - | 41 | 0 | - | - | - |
| rs3734078 | rs58045934 | 11 | 129,772,013 | 130,644,486 | 0,87 | -1.6628 | - | 41 | 0 | - | - | - |
| rs61908863 | rs34226067 | 11 | 134,416,490 | 134,616,366 | 0,20 | -1.9277 | - | 0 | - | - | - | - |
| rs521040 | rs6488433 | 12 | 10,147,850 | 11,768,462 | 1,62 | -1.7581 | - | 186 | 0 | - | - | - |
| rs1806201 | rs79974352 | 12 | 13,717,508 | 14,930,869 | 1,21 | -1.9018 | - | 40 | 0 | - | - | - |
| rs1886449 | rs76588355 | 13 | 73,932,114 | 74,797,460 | 0,87 | -1.7232 | - | 3 | 0 | - | - | - |
| rs283956 | rs76661894 | 13 | 77,117,984 | 79,330,073 | 2,21 | -1.8628 | - | 27 | 0 | - | - | - |
| rs10144623 | rs10137285 | 14 | 31,242,086 | 34,049,850 | 2,81 | -1.5711 | *COCH* | 64 | 1 | 0 | - | - |
| rs1837763 | rs77968073 | 15 | 71,707,375 | 73,236,886 | 1,53 | -1.4074 | - | 50 | 0 | - | - | - |
| rs351175 | rs75192007 | 15 | 74,528,985 | 77,518,629 | 2,99 | -1.7094 | - | 128 | 6 | 2 | 0 | - |
| rs117104971 | rs117140491 | 15 | 78,018,233 | 78,524,231 | 0,51 | -1.7302 | *CIB2* | 19 | 0 | - | - | - |
| rs78641696 | rs448653 | 16 | 1 | 1,081,348 | 1,08 | -1.0024 | - | 82 | 3 | 0 | - | - |
| rs17562963 | rs8044409 | 16 | 7,452,583 | 7,715,577 | 0,26 | -1.5463 | - | 5 | 1 | 0 | - | - |
| rs11077287 | rs74569016 | 16 | 8,337,001 | 9,781,606 | 1,44 | 0.5677 | - | 85 | 1 | 0 | - | - |
| rs138769038 | rs16942334 | 18 | 23,381,462 | 24,026,191 | 0,64 | -1.8843 | - | 5 | 1 | 0 | - | - |
| rs117246954 | rs117483780 | 18 | 77,241,715 | 78,077,248 | 0,84 | 0.1636 | - | 40 | 0 | - | - | - |
| rs4807391 | rs10415208 | 19 | 3,054,567 | 4,496,046 | 1,44 | -1.0559 | *GIPC3* | 230 | 1 | 1 | 0 | - |
| rs2250656 | rs1423096 | 19 | 6,718,534 | 7,739,177 | 1,02 | -1.9221 | - | 191 | 1 | 0 |  | - |
| rs1644731 | rs116049570 | 19 | 10,131,999 | 11,574,443 | 1,44 | -1.6460 | *S1PR2* | 196 | 2 | 2 | 1 | *RAVER1* c.2002G>A |
| rs1401828 | rs2144936 | 20 | 4,977,788 | 6,118,980 | 1,14 | -1.0977 | - | 51 | 1 | 0 | - | - |
| rs56055585 | rs4811952 | 20 | 56,296,576 | 56,583,939 | 0,29 | -1.7923 | *-* | 0 | - | - | - | *-* |
| rs2093140 | rs55872076 | 20 | 59,692,266 | 63,025,520 | 3,33 | -0.4991 | *OSBPL2* | 415 | 7 | 1 | 0 | *-* |
| rs1726175 | rs4141087 | X | 1 | 26,252,686 | 26,25 | 0.1244 | *SMPX* | 240 | 0 | - | - | - |
| rs4271113 | rs4239965 | X | 34,892,503 | 47,244,180 | 12,35 | -0.0783 | - | 82 | 2 | 1 | 1 | *BCOR* c.3407G>A |

Genomic positions are according to the UCSC Genome Browser, GRCh37/hg19 (https://genome.ucsc.edu/). Coverage of all exons and exon-intron boundaries of the ten known deafness genes located in one of the regions not excluded by linkage analysis, was manually checked to be at least 10x. Sanger sequencing was performed for regions with a lower coverage, which did not reveal any rare variants (allele frequency ≤ 0.005). VUS, variant of uncertain significance. ^a^Total number of WES variants shared by subject II:7 and III:8 of family W15-0551. ^b^Based on population frequency data of the dbSNP database and our in-house database. The latter contained WES data of 13314 individual, the vast majority of Dutch origin, affected by a large number of different diseases (including 810 subjects with HI). ^c^Based on variant classification guidelines by the Association for Clinical Genetic Science and the Dutch Society of Clinical Genetic Laboratory Specialists.(Wallis et al. 2013) ^d^Variants are listed in Table S3

**Table S3. Variants in region 73,739,831-96,400,830 of chromosome 6.**

| **Gene** | **RefSeq ID** | **Variant** | **dB SNP MAF (%)** | **In-house MAF (%)** | **ExAC MAF (%)** | **GnomAD-G MAF (%)** | **PhyloP** | **CADD** | **Predicted Effect on Splicing^a^** | **FPKM cochlea^b^** |
| --- | --- | --- | --- | --- | --- | --- | --- | --- | --- | --- |
| *SLC17A5^c^* | NM_012434.4 | g. 74310232del  c.1260-68del (p.?) | 0.000 | 0.000 | 0.00 | 0.57 | -0.993 | 0.799 | No effect on acceptor site; no novel splice site predicted | 2.67-4.77 |
| *PM20D2^d^* | NM_001010853.2 | g.89863059A>G  c.757+155A>G (p.?) | 0.119 | 0.250 | 0.00 | 0.268 | 0.004 | 4.118 | None | 0.18-0.85 |
| *MDN1^e^* | NM_014611.2 | g.90486180G>A  c.1821+139C>T (p.?) | 0.100 | 0.199 | 0.00 | 0.200 | -0.639 | 3.178 | None | 12.78-13.30 |

^a^ For prediction of a potential effect on splicing, sequence variants have been assessed by SpliceSiteFinder-like, MaxEntScan, NNSPLICE, GeneSplicer, and Human Splicing Finder as available in Alamut Visual (v 2.10, Interactive Biosoftware)

^b^ Values according to Schrauwen et al. Hear Res. 2016;333:266-274.

^c^ In the Mouse Genome Informatics (MGI) database a hearing phenotype is reported to be associated with a defect of this gene. Recessive mutations are associated with neurodevelopmental defects (OMIM #0269920, #604322) for which no hearing phenotype is reported.

^d^ No mouse mutants reported in the MGI or IMPC databases.

^e^ A homozygous defect, lethal at the preweaning stage is reported in the IMPC database.

CADD: via in-house pipeline according to http://cadd.gs.washington.edu/

IMPC, International Mouse Phenotyping consortium (http://www.mousephenotype.org/)

MAF, minor allele frequency

MGI, Mouse Genome Informatics (http://www.informatics.jax.org/)

Genomic positions are according to GRCh37/hg19

**Table S4a. Evaluation of candidate variants from WES in family 63136**

| **Gene** | **Variant Genomic** | **Variant cDNA** | **Transcript ID** | **Variant protein** | **In-house MAF (%)** | **GnomAD-E NFE MAF (%)** | **GnomAD-G NFE MAF (%)** | **CADD** | **SIFT** | **PPH2** | **Mutation Taster** |
| --- | --- | --- | --- | --- | --- | --- | --- | --- | --- | --- | --- |
| *ADRA2A* | chr 10:g.112,838,909G>C | c.1155G>C | NM_000681.3 | p.Lys385Asn | 0.016 | 0.025 | 0.060 | 17.36 ^p^ | 0.05 ^p^ | 0.978 ^p^ | Deleterious |
| *CAND2* | chr3:g.12,867,119G>A | c.3191G>A | NM_001162499.1 | p.Arg1064Gln | 0.008 | 0.014 | 0.020 | 31.00 ^p^ | 0.22 | 0.905 ^p^ | Deleterious |
| *INTS1* | chr7:g.1,526,355G>A | c.2882C>T | NM_001080453.2 | p.Pro961Leu | -1.000 | 0.001 | -1.000 | 15.88 ^p^ | 0.08 | 0.533 ^p^ | Deleterious |
| *TTYH3* | chr7:g.2,689,245C>T | c.767C>T | NM_025250.2 | p.Ala256Val | 0.020 | 0.011 | 0.007 | 21.70 ^p^ | 0.31 | 0.627 ^p^ | Polymorphism |
| *GRM3* | chr7:g.86,493,622C>T | c.2591C>T | NM_000840.2 | p.Thr864Met | 0.004 | 0.004 | -1.000 | 24.40 ^p^ | 0.00 ^p^ | 0.939 ^p^ | Deleterious |

For none of the variants, an effect on splicing was predicted by SpliceSiteFinder-like, MaxEntScan, NNSPLICE, GeneSplicer, and Human Splicing Finder as available in Alamut Visual (v 2.10, Interactive Biosoftware)

CADD: via in-house pipeline according to http://cadd.gs.washington.edu/

SIFT, PPH2 (PolyPhen-2) and Mutation Taster were employed via Alamut Visual (v 2.10)

^p^ predicted to be pathogenic

Genomic positions are according to GRCh37/hg19

MAF, minor allele frequency

**Table S4b. Evaluation of candidate genes from WES in family 63136**

| **Gene** | **HI phenotype in mouse mutants** | **FPKM in RNA of adult cochlea^a^** | **Expression (SHIELD database)** |
| --- | --- | --- | --- |
| *ADRA2A* | No HI phenotype (MGI, IMPC) | 0.041-0.457 | Low, in nonsensory cells of developing organ of Corti |
| *CAND2* | No HI phenotype (MGI, IMPC) | 0.311-1.419 | In developing organ of Corti |
| *INTS1* | No HI phenotype (MGI), no phenotype data (IMPC) | 0.288-1.201 | Low in developing organ of Corti and in spiral ganglia |
| *TTYH3* | no phenotypic data (MGI, IMPC) | 0.738-3.228 | In developing organ of Corti and in spiral ganglia |
| *GRM3* | No HI reported (MGI, IMPC) | 0.025-0.112 | In nonsensory cells of developing organ of Corti, low in spiral ganglia |

Defects in only INTS1 are associated with a Mendelian disorder, a rare recessive neurodevelopmental syndrome (OMIM). (https://thebiogrid.org/)

IMPC, International Mouse Phenotyping consortium (http://www.mousephenotype.org/)

MGI, Mouse Genome Informatics (http://www.informatics.jax.org/)

^a^ Values according to Schrauwen et al. Hear Res. 2016;333:266-274

**Table S5. Primer sequences and PCR conditions**

| Target | Primer | Oligonucleotides | Size (bp) | Annealing Temperature (°C) |
| --- | --- | --- | --- | --- |
| *LMX1A* exon 3 | Forward | gttctttcccaggagtgcg | 299 | 58 |
|  | Reverse | tgtatgaagaggggcgctag |  |  |
| *LMX1A* exon 4 | Forward | tggttgattagcaggagttcc | 461 | 58 |
|  | Reverse | ctgaagggaagttctgctcg |  |  |
| *LMX1A* exon 5 | Forward | tgaatgactcaactgaatgcttc | 500 | 58 |
|  | Reverse | agggaagaaacaatgcagacag |  |  |
| *LMX1A* exon 6 | Forward | tgctctcatacataaagccatcc | 376 | 58 |
|  | Reverse | caatgcccactgagatccca |  |  |
| *LMX1A* exon 7 | Forward | aggcaagagaggagaccaag | 576 | 58 |
|  | Reverse | acctggcagcaagtacaatg |  |  |
| *LMX1A* exon 8 | Forward | tggccactgttgctccttta | 395 | 58 |
|  | Reverse | ccctctcacaagttcctcgt |  |  |
| *LMX1A* exon 9 | Forward | ggaatcaggacccagggaaa | 347 | 58 |
|  | Reverse | tggaacttcggtgagcatct |  |  |
| *LMX1A* exon 10 | Forward | cagctgaatatcctagcactga | 395 | 58 |
|  | Reverse | GGAAATGCTGAGCTACACCA |  |  |
| *LMX1A* mRNA exons 3-4 | Forward | AGAACTTCCAAAGCGCGATC | 105 | 60 |
|  | Reverse | TCCAAGATGACCCGCTGAC |  |  |
| *LMX1A* mRNA exons 4-5 | Forward | TCTACCGGGACAAGAAGCTG | 111 | 60 |
|  | Reverse | GGGCCCGCATAACAAACTC |  |  |
| *LMX1A* mRNA exons 6-7 | Forward | CTCAACAGAGGCGAGCATTC | 97 | 60 |
|  | Reverse | ACACTCAGCCCTGTCTCTG |  |  |
| *MYO6* c.2417-1758T>G | Forward | ccattcatttgtggactgtg | 283 | 58 |
|  | Reverse | tgaaatgtgaataggtatgtcctg |  |  |
| *NR5A2* exon 8 | Forward | AATGTGTAACTACCCGCAGC | 396 | 58 |
|  | Reverse | AGCCTTTGATTCACAGTTTGC |  |  |
| *NRXN1* exon 2 | Forward | CCAGCTCAAGACTCGCAGC | 300 | 58 |
|  | Reverse | CCGACGAAAAGGCCGCTG |  |  |
| *PEX13* exon 1 | Forward | GTTGTGTCTTACGCTCCAGG | 294 | 58 |
|  | Reverse | ggttgggtattggttaaagggg |  |  |
| *CD164* exon 3 | Forward | tttgggtttgatttcatagagc | 264 | 58 |
|  | Reverse | ctgaaacaaggctttctgagg |  |  |
| *FZD1* exon 1 | Forward | CTTCGTGGGGCTTAACAACG | 273 | 58 |
|  | Reverse | CTGCGTTCCCACTGGTCC |  |  |
| *RAVER1* exon 11 | Forward | acaaagggctgggtactaca | 280 | 58 |
|  | Reverse | ggttacagagccccggtg |  |  |
| *BCOR* exon 8 | Forward | CACCGTGATCCCTTTGAAGC | 248 | 58 |
|  | Reverse | ggctcacCTTTAGAGACTCGT |  |  |
| *COL11A1* exon 26 | Forward | tgaattgaagccagtgactcag | 370 | 58 |
|  | Reverse | agtttccacaaaagccaccg |  |  |
| *COL11A1* exon 56 | Forward | tgctgtttttcagtattctaagagg | 657 | 58 |
|  | Reverse | gaaagtaaaatatgggagcacattag |  |  |
| *PNPT1* exon 7 | Forward | tacaaagcccctgcttttagc | 397 | 58 |
|  | Reverse | gccatatgccattgctgtaac |  |  |
| *PNPT1* exon 9 | Forward | gaaatcaaggtggatctatcactaag | 398 | 58 |
|  | Reverse | tccatgggaagtttctctcc |  |  |
| *COCH* exon 2 | Forward | ATCAGTCACCATGTCCGCAG | 497 | 58 |
|  | Reverse | cttcctcgacctcctgctg |  |  |
| *GIPC3* exon 1 | Forward | cctgtccctgtccttatttg | 746 | 58 |
|  | Reverse | gctagtcctaagacctgccc |  |  |

Primers for amplification of exons and exon-intron boundaries were designed with Primer3Plus (http://www.bioinformatics.nl/cgi-bin/primer3plus/primer3plus.cgi). The following reference sequences were used: *LMX1A*, NM_177398.3; *MYO6*, NM_004999.3; *NR5A2*, NM_205860.2; *NRXN1*, NM_004801.5; *PEX13*, NM_002618.3*; CD164*, NM_006016.5; *FZD1*, NM_003505.1; *RAVER1*, NM_133452.2; *BCOR*, NM_001123385.1; *COL11A1*, NM_001854.3; *PNPT1*, NM_033109.4; *COCH*, NM_001135058.1; *GIPC3*, NM_133261.2. Amplification by PCR was performed under standard conditions. PCR fragments were purified with ExoI/FastAP (Thermo Fisher Scientific, Waltham, MA, USA), in accordance with manufacturers’ protocols. Sequence analysis was performed with the ABI PRISM BigDye Terminator Cycle Sequencing v.2.0 Ready Reaction kit and analyzed with the ABI PRISM 3730 DNA analyzer or the 3130 Genetic Analyzer (Applied Biosystems, Foster City, CA, USA). A possibly deleterious effect of the identified variants on the proteins and splicing was predicted with Alamut Visual version 2.7.1 (Interactive Biosoftware, Rouen, France).

**Table S6. Vestibular assessment**

| Family | Subject | Velocity Step Test | | | | | | Caloric irrigation | | | | Conclusion |
| --- | --- | --- | --- | --- | --- | --- | --- | --- | --- | --- | --- | --- |
|  |  | **to right** | | | **to left** | | | **warm [^0^/s]** | | **cold [^0^/s]** | |  |
|  |  | **gain [%]** | **v(max) [^0^/s]** | **τ [s]** | **gain [%]** | **v(max) [^0^/s]** | **τ [s]** | **right** | **left** | **right** | **left** |  |
|  |  | [.33-.72] | [30-65] | [11-26] | [.33-.72] | [30-65] | [11-26] | [10-52] | | [7-31] | |  |
| W15-0551 | II:7 | 0,65 | 58 | ***1*** | 0,57 | 51 | ***1*** | ***0*** | ***2*** | ***2*** | ***2*** | Bilateral areflexia |
|  | III:8 | 0,44 | 40 | ***7*** | 0,48 | 43 | ***9*** | ***5*** | ***4*** | ***6*** | ***7*** | Bilateral hyporeflexia |
| 63136 | II:3 | - | - | - | - | - | - | ***6*** | ***2*** | ***3*** | ***1*** | Asymmetric hyporeflexia to areflexia to the detriment of the left vestibulum |

| Family | Subject | vHIT, gain [%] | | | | | | cVEMP, threshold [dBHL] | |
| --- | --- | --- | --- | --- | --- | --- | --- | --- | --- |
|  |  | **right** | | | **left** | | | **right** | **left** |
|  |  | **h-SCC** | **a-SCC** | **p-SCC** | **h-SCC** | **a-SCC** | **p-SCC** |  |  |
|  |  | [> 0.80] | [> 0.70] | [> 0.70] | [> 0.80] | [> 0.70] | [> 0.70] |  |  |
|  |  | 0,94 |  |  |  |  |  |  |  |
| W15-0551 | II:7 |  | 0,83 | ***0,57*** | 0,89 | 0,96 | ***0,40*** | n.r. | n.r. |
|  | III:8 | 0,99 | 1,01 | 1,00 | 1,01 | 1,01 | 1,00 | n.r. | n.r. |

Vestibular data of test results for caloric irrigation (n=3), rotary chair testing (velocity-step-test, VST), video head impulse testing (vHIT) for all semicircular canals (horizontal: h-SCC; anterior: a-SCC and posterior: p-SCC), and cervical vestibular evoked myogenic potential testing (cVEMP) of two subjects. Note that results of index case family 63136 (II:3) are only based on limited retrospective data.

Normative values are shown between brackets; gain in percentage; v(max): maximum nystagmus velocity at stop in degrees/sec; τ (‘tau’): time constant in seconds; n.r.: no response up to 100 dBHL (deviant values are shown in bold italic).

**References**

Beery KE, Buktenica NA, N.A. B (2010) The Beery-Buktenica developmental test of visual-motor integration: Administration, scoring, and teaching manual MN: NSC Pearson, Minneapolis

Nasreddine ZS et al. (2005) The Montreal Cognitive Assessment, MoCA: a brief screening tool for mild cognitive impairment Journal of the American Geriatrics Society 53:695-699 doi:10.1111/j.1532-5415.2005.53221.x

Schmand B, Lindeboom J, van Harskamp F (1992) Nederlandse leestest voor volwassenen. . Swets en Zeitlinger, Lisse

Schmidt M (1996) Rey auditory verbal learning test: A handbook. Western Psychological Services, Los Angeles, CA

Schmitz-Hubsch T et al. (2006) Scale for the assessment and rating of ataxia: development of a new clinical scale Neurology 66:1717-1720 doi:10.1212/01.wnl.0000219042.60538.92

Wallis Y et al. (2013) Practice Guidelines for the Evaluation of Pathogenicity and the Reporting of Sequence Variants in Clinical Molecular Genetics. ACGS /VGKL

Wechsler D (1991) The Wechsler intelligence scale for children. TX: The Psychological Corporation, San Antonio
